# A Self-Adaptive Model-Based Wi-Fi Indoor Localization Method

**DOI:** 10.3390/s16122074

**Published:** 2016-12-06

**Authors:** Jure Tuta, Matjaz B. Juric

**Affiliations:** Faculty of Computer and Information Science, University of Ljubljana, Vecna pot 113, SI-1001 Ljubljana, Slovenia; matjaz.juric@fri.uni-lj.si

**Keywords:** indoor positioning, Wi-Fi localization, propagation model, self-adaptive, received signal strength (RSS)

## Abstract

This paper presents a novel method for indoor localization, developed with the main aim of making it useful for real-world deployments. Many indoor localization methods exist, yet they have several disadvantages in real-world deployments—some are static, which is not suitable for long-term usage; some require costly human recalibration procedures; and others require special hardware such as Wi-Fi anchors and transponders. Our method is self-calibrating and self-adaptive thus maintenance free and based on Wi-Fi only. We have employed two well-known propagation models—free space path loss and ITU models—which we have extended with additional parameters for better propagation simulation. Our self-calibrating procedure utilizes one propagation model to infer parameters of the space and the other to simulate the propagation of the signal without requiring any additional hardware beside Wi-Fi access points, which is suitable for real-world usage. Our method is also one of the few model-based Wi-Fi only self-adaptive approaches that do not require the mobile terminal to be in the access-point mode. The only input requirements of the method are Wi-Fi access point positions, and positions and properties of the walls. Our method has been evaluated in single- and multi-room environments, with measured mean error of 2–3 and 3–4 m, respectively, which is similar to existing methods. The evaluation has proven that usable localization accuracy can be achieved in real-world environments solely by the proposed Wi-Fi method that relies on simple hardware and software requirements.

## 1. Introduction

Localization of devices is becoming increasingly important for the modern services that ease people’s lives. Outdoor localization has been studied deeply and worldwide systems for determination of position were built. Global Positioning System (GPS) and GLObal NAvigation Satellite System (GLONASS) were built to provide accurate location information in the outdoors. Reliable indoor localization is a must for the development of the future of the Internet of things (IoT). Sensors that are part of the IoT can benefit from accurate indoor location determination. Applications such as autonomous transportation systems in factories, temperature sensors of the IoT heating systems and robotic vacuum cleaners in residential homes will be able to perform better if object’s indoor location can be determined accurately. Indoor localization also opens new possibilities for digital products and services. Location-aware mobile applications that are recently more and more in use would benefit from indoor location information. The infrastructure for IEEE 802.11 technologies is widely available in residential and business buildings. Majority of indoor locations nowadays have Wi-Fi coverage, therefore there is much effort put into researching how to use this infrastructure for location determination. 

Current view of the future of the IoT predicts a variety of devices and appliances working together and exchanging information. Because of their variety, they do not have many common points, except one—they are all connected via some sort of wireless communication to the Internet. As not all devices are moved indoors, localization systems that fuse Wi-Fi localization data with inertial measurement unit (IMU) sensors cannot provide definite answer to indoor localization for IoT. Similar can be said for different techniques levering the visual information, as can be seen in [1]. There have been many different approaches to indoor localization researched over the last decade [2]. We could divide them into two groups: one group consists of methods heavily influenced by offline calibration procedure called fingerprinting, and the other group includes methods based on physical models while using principles of trilateration, triangulation or proximity [3]. 

Fingerprinting localization algorithms consist of offline stage in which fingerprinting of the RSS in the area of interest has to be performed. In the localization stage, algorithms would search for the best match in the database. Researchers analyzing different wireless signals for indoor localization agree that due to temporal changes in Wi-Fi signal [4] emitted by the access points (AP) no parameter calculated from measurements can be assumed as fixed [5,6].

Research done by Chan Chiang et al. [7] showed a fingerprinting algorithm, which was improved by utilizing clusters of mobile terminals. Nodes inside the cluster are located with fingerprinting algorithm; knowledge about the clusters of mobile terminals, discovered opportunistically by ZigBee is then used to collaboratively correct the error of the algorithm in location estimation. A similar approach by Kim et al. [8] used anonymous mobile users to automatically collect fingerprinting data and progressively build a precise radio map on the server. These approaches are not best-fitted for real-world applications, as mobile users have to agree and collaborate in the fingerprinting step. More recently, solutions that fuse data from IMU and Wi-Fi localization systems have become popular topic in the indoor localization research. Typical IMU consists of multi-axis accelerometer, gyroscope and magnetometer. For example, work done by Alverat, Alonso and Trivino [9] proposed an activity recognition system that uses Wi-Fi based location system combined with accelerometers for body posture recognition. Their Wi-Fi localization system uses a fuzzy rule based classifier that was generated on a training set obtained by fingerprinting the environment. A similar approach was used by Alonso et al. [10], although in the domain of small scale movements. Another often-used method for fusion is Kalman filter, as can be seen in [11,12,13]. We can find less frequent fingerprinting approaches, e.g., non RSSI based methods and decision-tree based fingerprinting algorithms, in [14,15].

Model-based approaches that use Wi-Fi RSS only to determine the location had to be developed for the cases where we do not have access to any other sensors and training sets cannot be obtained. Thus, it is not surprising that there was a significant research effort put into localization of specific APs in a non-surveyed environment. These methods can be used for discovering attacks on wireless networks and discovering Wi-Fi jammers [16,17]. Results from these research shows that it is possible to build model-based localization technique even when an access point we want to localize does not cooperate in the localization procedure. A case that is addressed during our research work is different as both the mobile terminal and the infrastructure have common goal in determining the location of the mobile terminal. 

Bisio et al. [18] proposed a method in which propagation map is not defined by means of sampling space but instead on pure physical models of finite-difference time-domain simulations. This approach did not properly address the effects of other devices and movable objects; thus, this method can be problematic in real life situations. Similar to authors of [10] in fingerprinting section, Olivera et al. [19] used probabilistic method to determine the position of a robot on a Wi-Fi map. Former authors chose modeled approach based on radio propagation model. They emphasized on selecting correct values for the parameters of the model. As in fingerprinting methods, in modeled approaches, there was also research done to utilize anonymous crowd to obtain samples. In the work done by Zhuang’s [20] team, these values were used to calculate parameters of the Wi-Fi propagation model. 

Some research has been devoted to identify improved metrics over RSS. Wu et al. [21] tried to develop methods to detect indoor human precedence with the usage of Wi-Fi signal only. They showed that channel state information (CSI) can be in some cases stable enough to develop a method that can detect stationary and moving people inside the rooms. Francisco and Martin [22] proposed their own metric for characterizing radio signal space for wireless device localization. 

We can find approaches that try to refine the model based approach to indoor localization. An example of such approach is [23], whose authors used points with well-defined location via Li-Fi emitters to calibrate parameters in the propagation models. In comparison to our proposed method, such approaches require additional infrastructure to be deployed to the building.

In the modeled approaches research, we can find usage of other sensors to augment Wi-Fi localization data in effort to provide better results. Work done by Zampella et al. [24] provided a fusion based approach, where modeled based Wi-Fi location was combined with pedestrian IMU data. On the other hand, Chiou et al. [13] used Kalman filter to fuse data from Wi-Fi propagation model and measurements of SNR data to obtain location information. This approach also uses additional hardware—reference points (Wi-Fi tags)—to build infrastructure for localization system. Indoor localization approach taken by Stubing [25] requires additional hardware—Wi-Fi transponders. These solutions are not generic. Fusion systems demand some other source of data rather than Wi-Fi signal which limits system’s potential for future use in the area of IoT.

Approaches that usually leverage knowledge about architectural aspects of the indoors are ray-tracing methods. Ray-tracing methods can be considered as a sub-group of model based approaches because they focus on directions, reflections and power propagation of signals emitted from Wi-Fi enabled devices. These approaches are usually computationally complex, because they calculate signal strengths at specific points due to direct and reflected paths. Examples of such approaches can be found in [26,27]. Some other research works present approaches aware of the indoor settling. Example of such work can be found in [28] where possible transitions between rooms are included into location determination, although this work addresses only determination of the room and not the exact location.

Paper by Tarrio et al. [29] considered real-life limitations; their goal has been minimizing the calibration procedures while improving the accuracy of localization. They proposed a positioning algorithm to calculate the position of a mobile node in an ad-hoc network from a set of distance estimations to the anchor nodes. Similarly, a paper written by Durmont and Corff [30] acknowledged the real-world limitations. They emphasized on environmental dynamic and problems of models, which often overlook these. Their algorithm is running online and does not require any offline calibration procedure. Propagation maps are estimated online using the data sent by the mobile device. Model-based approaches like [30,31] use signals emitted by mobile terminal to determine location, but this usually means that mobile terminal must be in AP mode. In real-world, this means that mobile terminal emits Wi-Fi beacons. Deploying such localization schemas to real-world environment (e.g., commercial building) would quickly saturate Wi-Fi channels and consequently reduce the speed of the Wi-Fi network. Nevertheless, we must also consider that the Wi-Fi adapter in AP mode has higher energy consumption than in the standard (Wi-Fi client) mode, which is very important for battery-powered devices. 

The paper most related to our research was written by Du Yang and Xiu [31], who proposed a modification of AP firmware to provide scanning feature to the APs. As findings presented in our paper are results from two-year-long research, our teams independently decided similar steps of modifying firmware. There are two fundamental differences between their approach and ours. Firstly, their team deployed the anchor points to infer current parameters of the propagation, which we have not, as we estimate the parameters from the communication between APs. Secondly, Wi-Fi devices can be in different modes. Their approach requires mobile terminal to be in AP mode, meaning that it emits beacon frames and other devices can connect to it. In our case, mobile terminal can be in standard (i.e., Wi-Fi client) mode in the network. 

In the beginning of 2016, Wi-Fi Alliance confirmed a new low power, long range Wi-Fi standard called HaLow that is defined as IEEE 802.11ah [32]. The new standard introduces a new frequency band, 900 MHz. Transfer speeds of the new band will be considerably lower, but the range of the signal will be extended. This new band is specially designed for variety of power-efficient use cases in the area of IoT, smart homes, connected cars, agriculture, healthcare and industry. The range of the new band is nearly twice as much as today’s Wi-Fi and is less susceptible to penetration loss when waves travel through the walls or other similar barriers. Therefore, it is even more important for the future to develop model-based indoor Wi-Fi localization system that will not need fingerprinting processes as they are time consuming and therefore costly to deploy in the real-world. Hardware that would support this standard is not currently available, therefore we focused on 2.4 GHz Wi-Fi band as it has bigger reach than the 5 GHz band. 

This paper proposes a novel approach to Wi-Fi localization with the main aim of delivering localization method that can be used in real-world situations. Our main contribution in comparison to existing research is purely model-based and self-adaptive localization method, without the need for fingerprinting, or fixing any parameters of the Wi-Fi propagation, as is often the case in existing solutions. We present a method that utilizes some of the well-known FSPL and ITU propagation models. As our research indicated that the introduction of another parameter would benefit the accuracy, propagation models were extended with the new parameter. Secondly, our presented method for inferring the parameters of the space without the need for additional hardware or special modes on the mobile terminal provides an effective method for system self-calibration to which many other propagation models can be applied. Thirdly, low hardware requirements in comparison to the other model-based methods (i.e., no anchor points, transponders, etc.) open the possibility for the method to be easily adopted and implemented by other researchers.

The reminder of this paper is divided into four sections. We summarize the goals for the method and explain the method in Section 2. In Section 3, we set the experimental parameters, describe the evaluation and showcase the results of our proposed localization method. We show the evidence how the signal strength between the two fixed positions changed in an eight-week experiment. In Section 4, we provide a discussion about the results, outline the problems of approaches that rely on measurements of RSS and discuss the result in comparison to other methods. We give conclusions and highlight future research directions in Section 5.

## 2. Proposed Localization Method 

Our goal was to develop a model that would be suited for real-world usage; each design decision was subjected to this goal. Motivation for the development of a new method was to address the difficult calibration procedures, static usage of parameters in propagation models and hardware requirements of existing methods. To accomplish this, we have set the following prerequisites:

**1.**  **Pure model-based Wi-Fi only approach**: As shown in Section 1 only pure model-based methods are suitable for big scale real-world deployments [5].
**2.**  **Self-calibrating operability**: Frequent recalibration method must be autonomous and should not require human intervention. Frequent recalibration insures method to be self-adapting to the changes of the indoors. Results from the experiments presented in Section 3.1 show us long-term temporal instability of the Wi-Fi signal, which confirms the claims in [4].
**3.**  **Awareness of architectural aspects**: Input data to our algorithm consist of information about access points and wall placements.
**4.**  **Applicability on widely available hardware**: We could maximize our chances for success by using advanced and costly equipment, which would provide more stable signals and reliable data readings, but we decided to develop methods on cheap and widely available hardware. More advanced mobile terminal equipment could also provide other information than received signal strength indicator (RSSI) (e.g., CSI), but this would limit the method usage to such devices.

**Figure 1:**
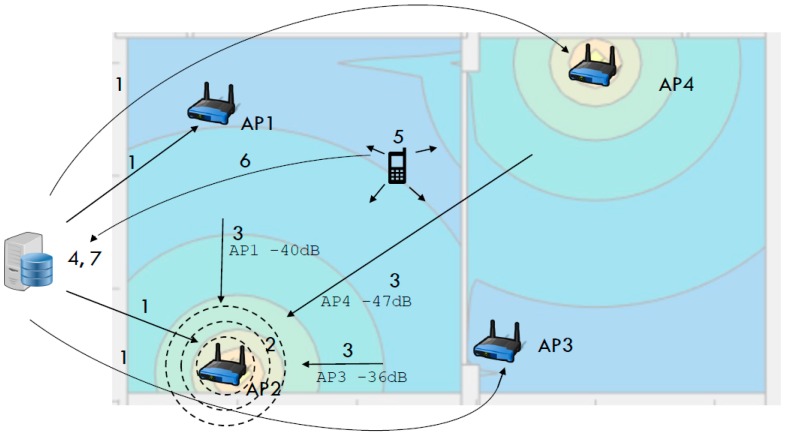
High-level overview and main steps in the proposed localization schema.

Our localization method can be divided into data acquisition stage, path loss modeling stage, propagation simulation stage and localization stage, which are presented in Figure 1. During the data acquisition stage the server periodically queries (Position 1 in Figure 1) each router for the survey (Position 2 in Figure 1) of the RSSI of the signals emitted from other access points (Position 3 in Figure 1). This information is used to infer the parameters of the space in the path loss modeling stage. After attenuation parameters are determined propagation for each AP can be simulated via selected propagation model (Position 4 in Figure 1). In localization stage, mobile terminal surveys the Wi-Fi spectrum for the APs in reach and determines RSSI for each of them (Position 5 in Figure 1). This information is then sent (Position 6 in Figure 1) to the localization server, which determines a point in the simulated propagation map that fits measured RSSIs (Position 7 in Figure 1). 

### 2.1. Data Acquisition Stage

**Figure 2:**
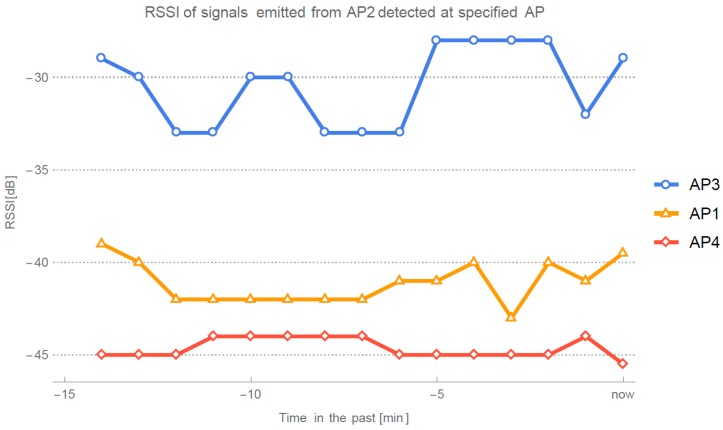
Example of signals emitted from AP2 detected at AP1, AP3 and AP4.

In data acquisition stage, localization server queries each AP for the results of the site survey. Results that list APs and their respective RSSI are then stored in the database on the server. Every time RRSI data from AP is acquired, new propagation parameters are calculated for the current setting. When calculating attenuation parameters for specific AP, database is queried for the measurements of the signal originating at this AP and detected by another AP. These data are then filtered by the median filter to eliminate signal outliers that can occur in the signal due to instability and variance of RSSI. Figure 2 shows an example of filtered RSSI data from which parameters are inferred.

IEEE 802.11 standard defines RSSI as a value to measure the relative signal strength. There is no standard definition of how the value is measured, thus values, scale and any correlation with the measured values of APs depend of manufacturer implementation [33]. Therefore, usage of different APs in this stage would make RSSI values incomparable. That is why all researches building model-based approaches with the help of parameters sensed at different APs use the same hardware for the APs as can be seen in [4,29,31]. This fact is also acknowledged during the development of our method. For this or any other similar method to be adopted by the general public, hardware manufacturers should standardize RSSI values. When deploying Wi-Fi infrastructure in a building (e.g., airport), APs are often of the same type, which solves the problem of non-standardized RSSI values. 

### 2.2. Path Loss Modeling Stage

We started with the logarithmic-distance path loss model for propagation-parameter estimation as it is often used as a baseline for model-based method development [13,31]. Path loss model can be expressed as:

(1) $$PL=PL_{0}+10\gamma \;\log_{10}\frac{d}{d_{0}}+\Delta$$

where PL is total path loss at distance d, $PL_{0}$ is total path loss at a distance $d_{0}$, γ is path loss exponent and Δ is variable accounting for variation of the mean and is often referred to as shadow fading [31]. Let us mark each entry into database with $RSSI_{j,i}$, where j is labeling $AP_{j}$ at which signal was measured and i stands for $AP_{i}$ from which measured signal was emitted. While identifying parameter $\gamma_{i}$ at the time t for the propagation of signal originating from the $AP_{i}$ we query the database for last h $RSSI_{m,i}$ and $RSSI_{n,i}$ collected in the interval δ, where m, n present pair of APs, which are not $AP_{i}$. Knowing the spatial positions of the access points we can obtain distances $d_{i,m}$ and $d_{i,n}$, which are distances between $AP_{i}$ and $AP_{m}$ or $AP_{n}$. Rewriting Equation (1) with introduced indexes yields an equation that we can use to determine $\gamma_{i}$.

(2) $$RSSI_{m,i}-RSSI_{n,i}=10\gamma_{i}\;\log_{10}\frac{d_{i,m}}{d_{i,n}}$$

Path loss exponent $\gamma_{i}$ for the access point $AP_{i}$ can then be calculated by utilizing least squares regression of Equation (2). Experimental evaluation, as presented in Section 3.4, showed that omitting measurements from the AP, which is positioned on the same wall as $AP_{i}$, would yield better accuracy. For example, parameters for the AP2 in Figure 1, calculated on the basis of measurements gathered at AP3 and AP4, would yield better performance than if measurements from AP1 were used. Difference in parameter estimation was appointed to the reflection of the wall on which transmitting AP is mounted. To account for this phenomenon, we have extended Log-distance path loss model with parameter $\beta_{i}$, which accounts for the effects in angle difference between direction of direct-signal-path and normal vector to a wall on which AP is mounted. Extended log-distance path model can be written as:

(3) $$RSSI_{m,i}-RSSI_{n,i}=10\gamma_{i}\;\log_{10}\frac{d_{i,m}}{d_{i,n}}+10\beta_{i}\;\log_{10}\frac{d_{i,m}}{d_{i,n}}\times \left(\alpha_{i,m}-\alpha_{i,n}\right)$$

where additional symbol introduced $\alpha_{i,j}$ is defined as:

(4) $$\alpha_{i,j}=\frac{∢\left(n_{i},s_{i,j}\right)}{\pi /2}$$

In Equation (4), $n_{i}$ represents normal vector to a plane defined by wall on which $AP_{i}$ is positioned, $s_{i,j}$ represents vector with direction of direct signal path between $AP_{i}$ and $AP_{j}$ and $∢\left(n_{i},s_{i,j}\right)$ denotes sharp angle between vectors $n_{i}$ and $s_{i,j}$ in radians. Normal vector can be calculated from data inputted to the method—information about APs and wall placement.

With Equation (3), we can use data from all measuring APs as input into least squares fitting to determine values $\gamma_{i}$ and $\beta_{i}$. This means we have N×(N−1)/2×h measuring data points to infer the parameters of signal propagation, where N is the number of AP in reach of $AP_{i}$. This results in overdetermined system of equations, in which usage of least squares regression finds parameters with best overall fit. 

Final form of the formula for inferring the parameters of propagation includes the effect of the walls. Method’s input includes the information about the placement of the APs and the walls, therefore it is trivial to calculate the number of walls between APs. List of the walls is the initial input into the method. The method contains a parameter, which describes the wall type. We use the values recommended by the European COST action 231 [34] for effects of a specific wall types on the propagation. Equation (5) represents the final formula for inferring the parameters of the propagation:

(5) $$\left(RSSI_{m,i}-W_{m,i}\right)-\left(RSSI_{n,i}-W_{m,i}\right)=10\gamma_{i}\;\log_{10}\frac{d_{i,m}}{d_{i,n}}+10\beta_{i}\;\log_{10}\frac{d_{i,m}}{d_{i,n}}\times \left(\alpha_{i,m}-\alpha_{i,n}\right)$$

where $W_{k,i}$ represents effects of walls between $AP_{i}$ and $AP_{k}$. To compute value $W_{k,i}$ method searches through the list of walls, which we have defined as the input parameter into the method. Method counts the number of walls between $AP_{i}$ and $AP_{k}$ grouped by their type. In the final stage, we apply the effect of each wall-type recommended by COST action 231 and then calculate the cumulative effect. If information about the wall types cannot be obtained default values for plaster or concrete wall can be used. In this case, we can expect bigger average localization error in the buildings with mixed wall types.

When calculating parameter $\gamma_{i}$ from Equations (2), (3) or (5), it is important that distance ratio $\frac{d_{i,m}}{d_{i,n}}$ is not close to 1. If $d_{i,m}\approx d_{i,n}$, than due to variations in Wi-Fi signal, we can get values $\gamma_{i}<0$, which would mean, that signal while traveling away from antenna gets stronger. Such situations occurred often in multi-room evaluation, where AP3 was positioned approximately equidistant from all other APs. To provide failback for such cases, we check if inferred $\gamma_{i}$ is lower than half of the power-loss factor in theoretical free space propagation ($\gamma_{i}<1$). In such case we, set $\gamma_{i}$ to the value proposed by ITU standard for commercial buildings $\left(\gamma_{i}=20\right)$. We have chosen fail-back parameter for commercial building instead the one for the office, as the office building power-loss exponent is bigger because there are usually more walls in the office buildings that the signal has to penetrate. Our method explicitly calculates the number of walls and their effect; therefore, we have chosen commercial buildings, which are similar but tend to have fewer dividing walls.

### 2.3. Propagation Simulation Stage

Knowing the calculated parameters of signal propagation, we chose a model, which was built to estimate the path loss inside a closed area for the propagation simulation. This is why we decided to use ITU indoor propagation model [35]. Model is developed and regularly updated by International Telecommunication Union, which is an agency of the United Nations. ITU model tries to account for reflections and diffractions caused by objects, channeling of energy, motions inside the room, multipath effects, etc. Their model provides guidance on indoor propagation over a frequency range from 300 MHz to 100 GHz. Basic model can be expressed as [35]:

(6) $$PL_{i,j}=20\;\log_{10}\left(f\right)+N\;\log_{10}\left(d_{i,j}\right)+L_{f}\left(n\right)-28$$

where $PL_{i,j}$ is total path loss of a signal originating in access point $AP_{i}$ at point j in dB, f is frequency in MHz, N is distance power loss exponent, $d_{i,j}$ denotes distance in meters between $AP_{i}$ and point j. $L_{f}\left(n\right)$ factor accounts for the loss between floors, which is currently the not focus of our research, therefore we omit its effect. ITU whitepaper gives predictions for power loss coefficients and other parameters for specific frequencies and building types. As we have measured power loss exponent in data acquisition stage of our method, we adopted ITU model by using our own measured values. 

Inclusion of the proposed parameter β and the effect of the walls into the ITU model results in Equation (7), which calculates power loss at a specific point j, in which $\alpha_{i,j}$ is defined by Equation (4). This time $s_{i,j}$ represents direction of a signal from $AP_{i}$ towards point j:

(7) $$PL_{i,j}=20\;\log_{10}\left(f\right)+\gamma_{i}\;\log_{10}\left(d_{i,j}\right)+\beta_{i}\times \alpha_{i,j}\times \log_{10}\left(d_{i,j}\right)+W_{i,j}-28$$

In propagation simulation stage, localization server computes the expected power losses inside the room for each AP in mesh pattern and constructs the radio propagation map. 

### 2.4. Localization Stage

In the localization stage, mobile terminal surveys available APs. On the mobile terminal we do not have a similar problem as in the data acquisition stage, where this information was unavailable to the end-user. Mobile terminals usually present RSSI information to the end-user in order to give the user the information about the signal strength. Even if this information is not available in numeric form, any applications that will leverage this usually have access to this information through the operating-system libraries. List of available APs is then sent to the localization server for further processing. 

To account for variations in RSSI, our method samples the RSSI 3 times, therefore we get a vector of 3 measurements of RSSI, labeled as $RSSI_{MT,i}$, for each access point $AP_{i}$. Mean value for each AP is then used for localization. Localization point is thus defined as:

(8) $$RSST_{MT}=\left\{\mathrm{Mean}\left[RSSI_{MT,1}\right],\mathrm{Mean}\left[RSSI_{MT,2}\right],\ldots \right\}$$

When determining position of a mobile terminal we cannot directly compare absolute values of estimated path loss values and readings from the mobile terminal, because they were collected on a different hardware. Additional losses in the mobile terminal can also be due to obstructions from people holding mobile devices, different materials from which mobile terminal (e.g., mobile phone) is made, different additional cases that are added to mobile terminals, etc. Assumption for the method development is that these effects are equal on all sampled signals during specific measurement. If we assume that the difference in RSSI readings between APs is a consequence of path loss model (Equations (3), (6) or (7)), we can assume that the difference between received powers in dB scale is the same in the simulator environment and in the real measured values. 

Model assumes that the most powerful measured reading is most the stable, therefore we subtract $\max\left(RSST_{MT}\right)$ reading from each $RSSI_{MT,i}$. Because we know from which $AP_{k}$ $\max\left(RSST_{MT}\right)$ originated, we subtract the value of simulated $PL_{k}$ from all $PL_{i}$ in each point of the map generated in the propagation simulation stage. Then we can get the position of the mobile terminal by finding the point in the map, which has the smallest error when comparing measured differences $RSSI_{MT,i}-RSSI_{MT,k}$ and simulated values $PL_{i}-PL_{k}$.

## 3. Results

This section presents the results obtained during our evaluations. First subsection defines the experimental parameters. Second subsection holds the detailed description of the evaluation space. In the third subsection we present the results from week-week-long experiment on variance of Wi-Fi RSS to further support our claims that only self-adaptive and self-calibrating methods are viable candidates for real-world deployments. Section 3.4 and Section 3.5 present the evaluation of the method in single- and multi-room environments, respectively. We decided to evaluate our method separately in single-room environment because of method’s new underlying propagation model. We wanted firstly to evaluate the method and its underlying model in an environment without additional variables (e.g., wall effects). After we obtained the model’s base performance in a single room, we continued the evaluation in a multi-room environment. 

### 3.1. Experimental Prerequisites

To eliminate the need for fingerprinting our method requires capability of the APs to survey the Wi-Fi channels and report RSSI of the neighboring APs. Majority of the APs in the market nowadays have this functionality built-in as it is an integral part of automatic determination of the most suitable channel for Wi-Fi communication. Usually information about the readings is not available to the end user. This is one of the reasons, besides its popularity, why we chose one of the most widely used wireless routers—Linksys WRT54GL. It has an open source third-party firmware called OpenWRT that exposes surveying information to the end-user. This is the biggest deviation from our development goal of real-world usability, but because this functionality is already built into the routers, it could easily be exposed by the manufacturers. For research purposes, OpenWRT is available for many APs in the market.

Surveying is a periodically occurring event. If its frequency is too high, this results in additional load on the Wi-Fi network, which is unwanted as it impacts the data-transfer performance on the wireless network. Too low frequency means changes in the Wi-Fi network we want to adopt will took too much time to become meaningful and effective. A closely related parameter is the number of historical surveys h our method will use. Choosing too many will have similar consequences as a too long period. Too little would result in higher influence of RSSI variance than desired. After elaborate testing, we have chosen 1 min as a scanning period and h=15 history points. This insures that changes due to long-term temporal instability of the Wi-Fi and changes in indoor space will have significant effect in less than 10 min, as they will be present in more than 50% of the data-points used for the calculation. Third important parameter for the data acquisition is the median filter window size. We chose windows of size 3 to eliminate high frequency notice from the data. 

During propagation simulation another important parameter is the density of the mesh for the propagation simulation. We choose value 0.2 m, as it provides mesh five times more dense than the density of the evaluation. 

**Figure 3:**
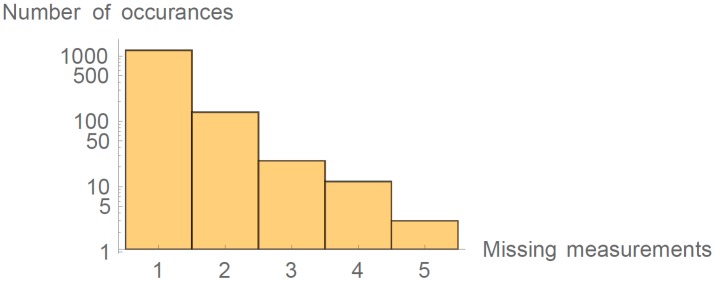
Missing detections of AP4 at AP2.

In real-world situations, one must predict a situation in which a specific AP preforms survey and does not detect one of the APs. We have analyzed eight weeks of surveying data between two APs. In Figure 3, we can see number of occurrences when a difference between two consecutive $RSSI_{j,i}$ between $AP_{i}$ and $AP_{j}$ in the database was more than 1 min, thus indicating that while $AP_{j}$ was reforming a survey it did not detect $AP_{i}$. Experiment lasted for 80,640 min; note the logarithmic scale on the y-axis. 

Results of this analysis show that the probability that $AP_{i}$ did not properly detect $AP_{j}$ while preforming the survey was 1.7%. To account for such situations, we advise to select 15 data points in the last 16 min. Experimental data show that the probability of such event occurring if choosing 15 history data points in the interval of 16 min is 0.34% per AP pair. During the evaluation presented in Section 3.4 and Section 3.5 the situation where two measurements would be missing in the window of 16 min did not occur. Implementation of the method predicted to continue without the missing points, which would result in the calculation with only 14 history points in the case of two missing measurements. Because our system is heavily overdetermined, the method would still result in location determination. 

### 3.2. Evaluation Environment

The main goal of our method development was to develop a method that is usable for real deployments and applicable to real-world situations; therefore, we had to set our evaluation procedure carefully. The selected evaluation environment was our office; part of one is organized as a community area, which exhibits many aspects of a typical living room. 

**Figure 4:**
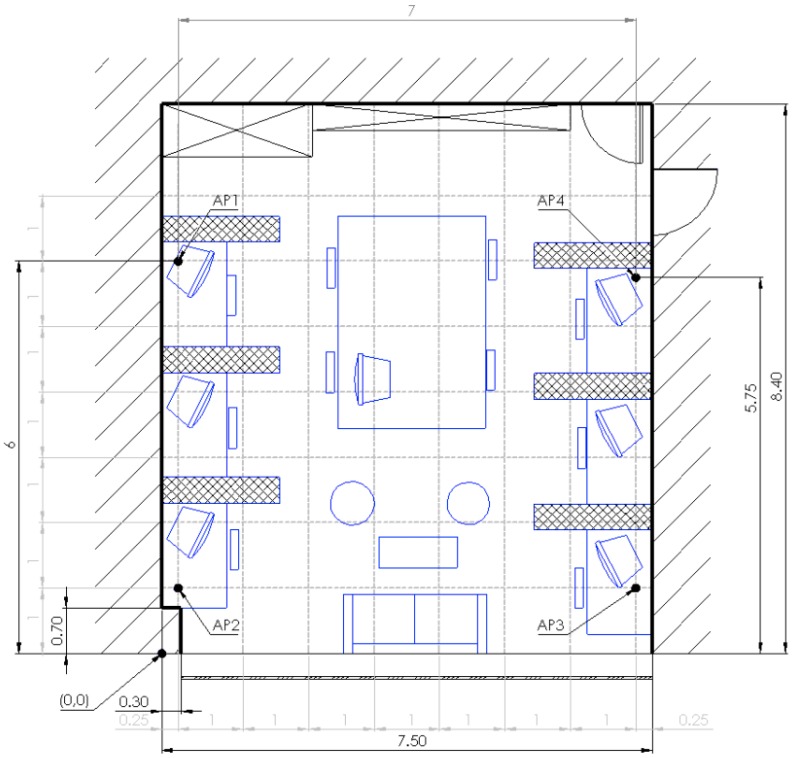
Evaluation environment in single room scenario.

Evaluation area is displayed in Figure 4. From the architectural point of view, left wall is thick concrete wall, right wall is made of plaster, wall at the top is filled with office storage closets from floor to ceiling, and at the bottom there is a huge window spanning the wall. This means that we have four different types of materials from which Wi-Fi signal can be reflected. Access points are positioned as represented in Figure 4. Thin dashed gray lines represent virtual mesh. At the crossings of the vertical and horizontal lines, except at the points in which APs are positioned, we evaluated our proposed method. All figures with results presented in this section have coordinate system matching the one in Figure 4, with axis origin in the point marked with “(0, 0)” annotation. In the case of AP4—due to spatial constraints—we could not position the AP at the crossing of the virtual mesh (position at (7.25, 6)) therefore we had to position it at (7.25, 5.75). Cross hatched blocks beside desks represent 1.6 m high divisors between workspaces.

Our office is usually full during workdays from 7:00 a.m. to 6:00 p.m.; during these times, more than 50 different Wi-Fi enabled devices enter or exit Wi-Fi range. Devices include laptops, tablets, mobile phones and smart watches. During the night or during the weekends at least 10 devices use Wi-Fi spectrum. To put heavier load on the system, we decided not to connect APs to the internet via Ethernet cable. APs were set in repeater mode and connected to the Internet via 5th AP. We have checked that 10 different APs are in range of our room (including 4 we used for evaluation). Evaluation environment also contained other than 2.4 GHz wireless signals, including 5 GHz Wi-Fi (IEEE 802.11 ac) and Bluetooth. Bluetooth speaker, which uses similar 2.4 GHz band, was present in the evaluation environment. To saturate channels even further, we configured all of the APs to use the same channel and maximize interference between them. For the mobile terminal, we chose Raspberry Pi 2 with simple USB dongle (commercial name WIPI) that provided Wi-Fi connectivity. We chose this wireless connectivity option as it is one of the simplest available: it has a single Wi-Fi PCB built antenna and low power—FCC testing reports maximal power below 11 dBm [36]. More advanced equipment used for the evaluation of our method could yield in even better results. 

### 3.3. Long Term Wi-Fi RSSI Variation

**Figure 5:**
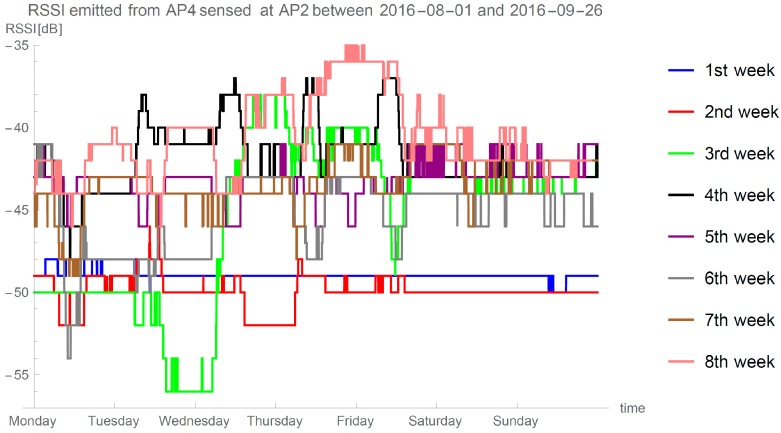
RSSI variance in long term experiment.

To support our claims and claims done in papers describing model-based approaches (e.g., [5,28,31]), i.e., that only self-calibrating and self-adaptive methods are suitable for real-world deployment, we designed a long term experiment in which we measured signals in our offices (described in Section 3.2) between two stationary APs. Figure 5 displays eight weeks of data of the signal emitted from AP4 and sensed at AP2. Measurements were taken every minute and median filter with width of 120 min is used to display the data. We can see that even with heavy filtering of the signal, it stills shows great variance through eight weeks.

### 3.4. Single-Room Evaluation

**Figure 6:**
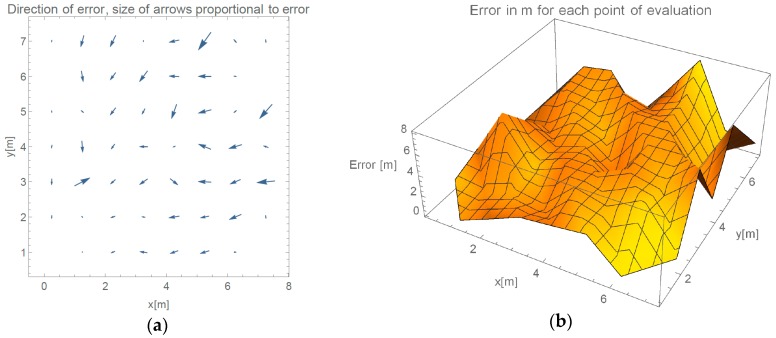
(**a**) Direction of errors in the case of using all available data for calculation of γ; and (**b**) value of localization error for each reference point if all RSSIs are used for calculation.

In this section, results from the evaluation of our proposed method are presented. We show results of progressive steps during the development of our method. We evaluated our method by localizing 52 reference locations as described in Section 3.2. In the case of using all 45 measured points with γ-only localization method, which implements propagation model given in Equation (2), more than 2/3 of the data points are heavily influenced by the effects of reflections under big angles. Figure 6a shows an overview of directions of the errors made by such method. In every point of the evaluation, we can see a point representing a direction of the error. Arrows size is proportional to the error they are representing. We can see that only the reference points close to APs were located accurately. Figure 6b shows the value of the localization error for each reference point.

As described, inferring γ from APs that are positioned on opposite walls yield better results. Mean and median errors are compared in Table 1. We can observe more than 25% improvement of average error if specific APs are omitted.

**Figure 7:**
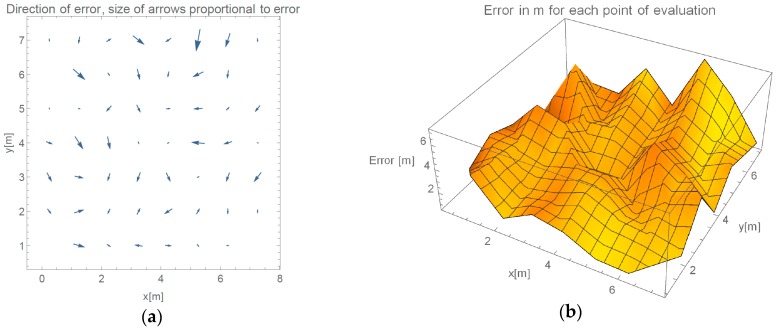
(**a**) Direction of errors in the case of omitting APs on the same wall for calculation of γ; and (**b**) value of localization error for each reference point, when omitting selected RSSIs.

Figure 7 presents measured error value and displays the direction of the errors when determining location in evaluated environment in the case we only use RSSI reported by APs on the opposite wall.

Sampling sequence started at point (1.25, 1) and continued along x-axis of the room towards (6.25, 1). Next, we collected data from (7.25, 2) towards (1.25, 2) and so on in left-to-right and right-to-left pattern. 

**Figure 8:**
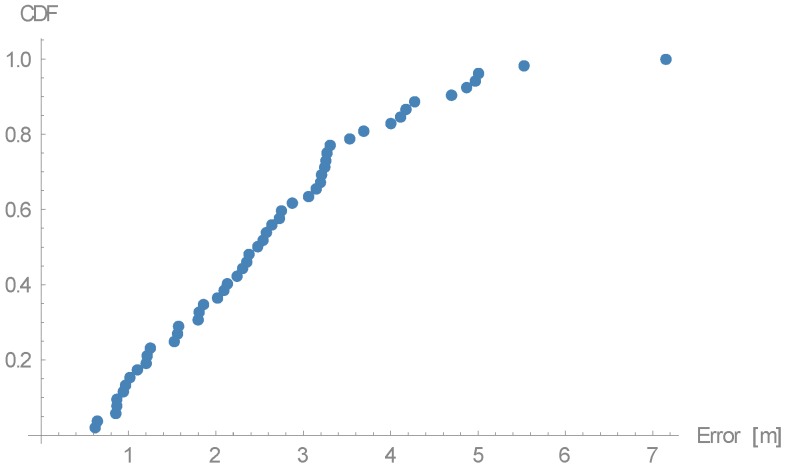
Cumulative Distribution Function (CDF) of the error of the 52 reference points.

Arrow lengths in Figure 6 and Figure 7 cannot be directly compared. Table 1 presents absolute difference in values between these two approaches. In Figure 7, we can see a more even distribution of errors through space. We can see one significant error made at evaluation position (5.25, 7). Error in this case is nearly 2 m bigger than in 95% of other measurements. This is also clearly shown in Cumulative Distribution Function (CDF) in Figure 8.

**Figure 9:**
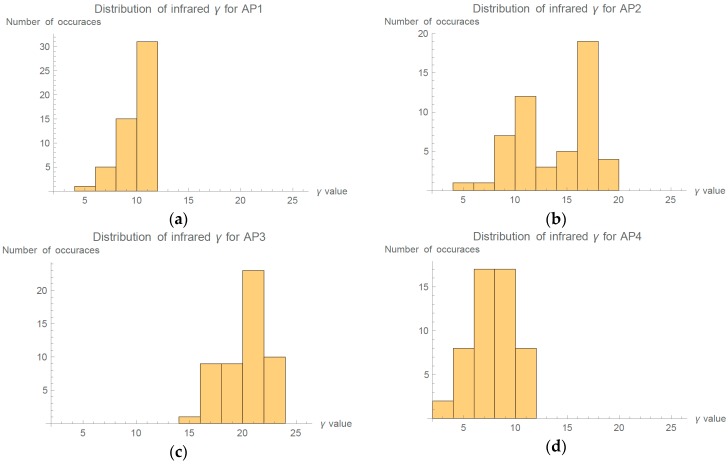
Histogram representing all 52 calculated γ for each AP during evaluation: (**a**) AP 1; (**b**) AP2; (**c**) AP3; and (**d**) AP4.

Path loss exponent is sometimes thought as a constant for all APs. This could be under the influence of misinterpreting different models, which propose specific values (e.g., COS231 line-of-sight model, ITU models, etc.). Figure 9 shows distribution of calculated γ parameters for each AP. In Figure 9a, we can see that AP1 was most stable as 88% of calculated γ span between 8 and 12. γ for AP3 and AP4 have expected symmetrical distributions. Interestingly, AP2 has clearly visible non-symmetrical distribution as its values reside in two groups. First major group spans values from 8 to 12, second from 14 to 20. 

**Figure 10:**
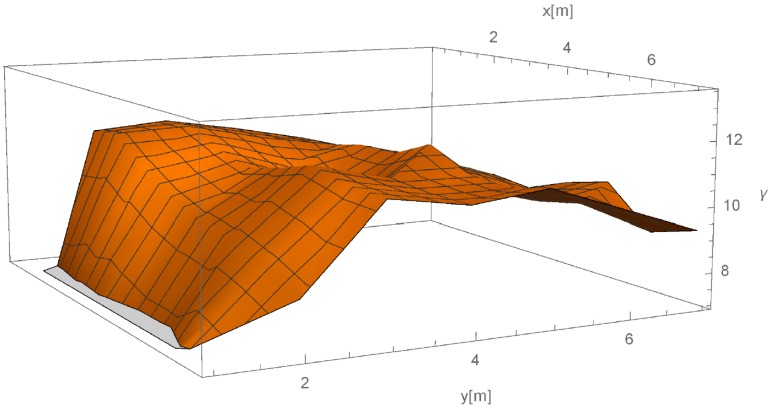
γ value calculated for every evaluation point.

We wanted to investigate the cause of non-symmetrical distribution of γ for the AP2, therefore we preformed temporal and spatial analysis. Figure 10 shows that first few measurements had extremely low γ value (below 10), which then suddenly rose to higher values and, during the length of experiment, slowly started to fall. Sudden change in Wi-Fi spectrum during the evaluation resulted in the non-symmetrical distribution of γ values in Figure 9.

**Figure 11:**
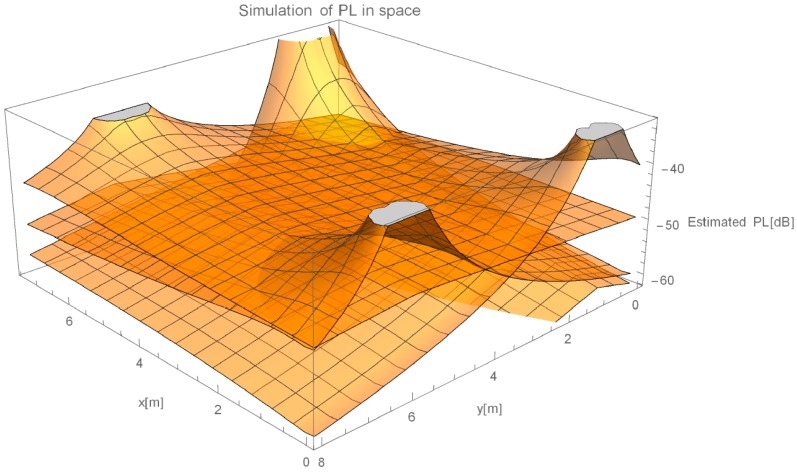
Output from propagation simulation stage of the method—simulation of path loss in the evaluating space emitted by four APs.

Introduction of parameter β (as defined by Equations (3) and (5) of our method) improved the simulation of signal propagation, which resulted in better accuracy of the results overall, although we can find one measurement whose location prediction significantly worsen. We can see improvements of prediction in Figure 11 in which output from the propagation simulation stage is shown. Figure 11 presents results from propagation simulation stage of our proposed method. It shows estimated path losses (z-axis) in space (x- and y-axes) for each of the four APs (four meshes). Therefore, by selecting a specific (x,y) in space, we can obtain information about estimated path losses from all 4 APs in the selected point. If we concentrate on the propagation emitting from AP2 (right in the figure), we can see that its propagation prediction at the AP1 (front middle in the figure) is lower than near AP4 (left in the figure) although AP4 is 50% further away from AP2 than AP1. This propagation model better describes the reality.

**Figure 12:**
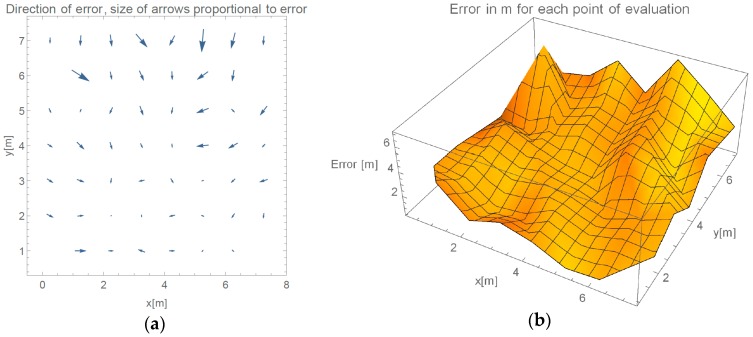
Errors of proposed method (**a**) Direction of errors; and (**b**) value of localization error for each reference point.

In Table 2, we can see how introduction of the parameter β improved the accuracy in comparison to γ-only model in which all APs were used. For easier comparison accuracy of the γ-only model with AP on the same wall omitted is kept. Table 2 shows we have achieved an even better improvement in comparison with γ-only model, which uses all APs. We can see 9% improvement in median error of the proposed method if we compare it to the γ-only method. Worsening of one of the localization can be seen in Figure 12 at position (1.25, 6), which is also one of the reasons for smaller improvement in the average error. 

**Figure 13:**
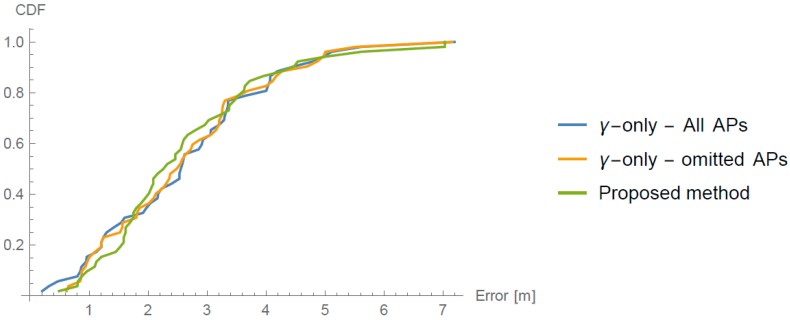
CDFs of the errors of all three discussed methods.

For easier comparison of the discussed models, Figure 13 presents CDFs of all three. From the graph, we can see that our proposed method is slightly worse than γ-only methods when the error is overall small. When the errors get bigger, our proposed method is better, with the already discussed exception.

### 3.5. Multi-Room Evaluation

**Figure 14:**
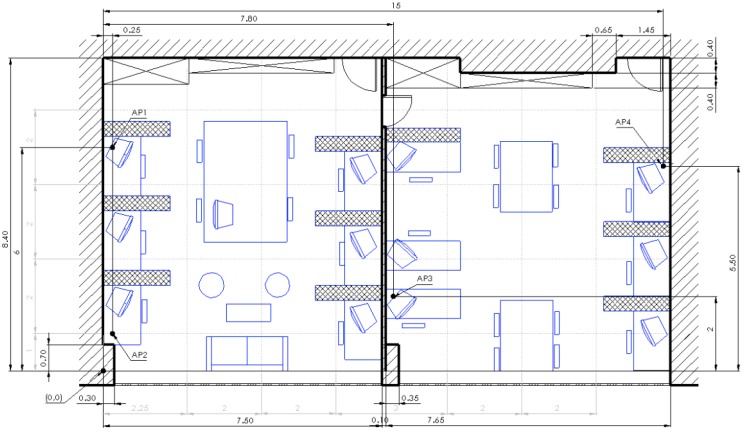
Evaluation environment—multi-room.

For our multi-room evaluation, we extended our evaluation into office next to ours. This time we purposely positioned APs in a non-symmetric manner. As the accuracy of the methods that infer parameters from the space depends greatly on the number of devices used—i.e., APs, anchors, Wi-Fi transponders, etc.—we decided that we will double the area but keep the same number of devices. Evaluation environment for multi-room setup is presented in Figure 14. The room exhibits fewer divisors between the worktables, smaller desks from which signal bounces and no community area. Because the dividing wall between the two offices is made from plaster, we used −4 dB as the value of the wall’s effect as proposed by COST 231 [34].

Evaluation in this environment consisted of 24 evaluation points. We performed evaluation on the crossings of virtual mesh with coordinates {(x,y);x∈{2.25,4.25,6.25,9.25,11.25,15.25},y∈{1,3,5,7}}. The error values of our single- and multi-room evaluation are presented in Table 3.

**Figure 15:**
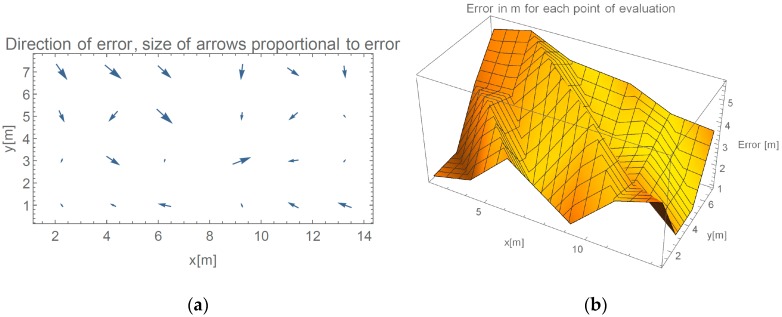
(**a**) Direction of errors in multi-room evaluation; and (**b**) value of localization error for each point.

In this evaluation, sampling sequence started in left room at point (2.25, 1)—i.e., lower left corner, and continued through left room in “left-to-right”, “right-to-left” pattern. After the last measurement in the left room at position (2.25, 7) there was a significant pause when we had to transfer our equipment into the second room, where sampling started at (13.25, 7) continuing in “top-to-bottom”, “bottom-to-top” pattern, finishing at point (9.25, 1). Figure 15 presents the direction and the value of the errors during multi-room evaluation.

## 4. Discussion

Our long-term experiment provides view into Wi-Fi propagation rarely seen in papers proposing localization methods. We can see a bit more stable signal during the weekends, when usually no people are present in the room. We observed at each selected time on any pair of the APs, at least 5 dB of variance. The 5 dB difference in RSSI at the distance of 8 m can mean that localization approaches that depends on values collected at some fixed point in time (e.g., fingerprinting methods, methods without autonomous user-intervention-free calibration procedure, etc.) can expect estimations between 5.5 and 12 m when locating a point 8 m away. Therefore, any fingerprinting based approach is prone to unacceptable errors in the long term deployment.

These findings confirm that only a model-based approach that can quickly adopt to the environmental changes and changes in the Wi-Fi spectrum can be used for real-world indoor localization deployment. Adoption speed is relative, changing values of parameters that have influence on it is balancing between stability of the results and sensitivity to changes. 

Another interesting observation from our long-term experiment is data levels during the 1st and 2nd week. These two weeks were the first weeks of August, when some of the people occupying desks in the office were on holidays. One would assume that smaller number of devices in the room would consequently mean less interference and channel usage, thus resulting in better signal (higher values in the figure), but we see the opposite situation. 

One of the concerns when designing the evaluation procedure was that sampling sequence order would have an effect on the measured error—i.e., when a few consequent evaluations points are sampled they are under the effect of some of the same measurements in 15 min time frame. Evaluation sampling of the 52 reference points took approximately one hour, as sampling three times RSSIs on the mobile terminal took approximately 30 s. Around 20 s was needed to determine the exact position of the next measurement. This resulted in every evaluation point having at least one different value in the history-data-point list used to infer the parameters. If there were influences of the sampling procedure, consequently sampled evaluation points would have similar error, which cannot be seen in Figure 7. Evaluation of the single-room and multi-room environment took place more than a week apart, with no changes to the system, except changing position of the two APs. Because our method, by its definition, utilizes only measurements gathered in last 16 min for location estimation our method can be used for long-time deployments. Results in accuracy of the two evaluations more than a week apart confirm these claims empirically. 

Strong evidence of robustness of our method is comparison of Figure 7 and Figure 10. In Figure 10, we can see there was a significant change in inferred the parameter γ while, in Figure 7, we do not see any significant change in the accuracy of the location estimates. From these data, we can see the method successfully adopted to a 4–5 dB change in the environmental parameter γ.

If we compare the best of the γ-only methods with our proposed method we do not see significant difference, but by definition of γ-only method we know, that it only works in the settings where measuring APs are positioned in a specific way. Although careful examination of CDF in Figure 13 or by looking at the average errors we do not see any significant changes, this is due to setting of the experiment. From the knowledge gathered when we transitioned from the first γ-only method to the second, which omitted some measurements, it is safe to say that the second γ-only method works well only due to the AP placement. In real-life deployments, we cannot assume that for each AP there will be two other APs in a desired position.

Multi-room experiment is interesting for one major reason. While doubling the area of possible localizations we did not double the number of devices, therefore the density of the Wi-Fi halved. Secondly, we did not create an ideal placement of the routers, where they would be placed on the corners of the area of interest. We made localization more challenging by positioning one of the routers in the lower middle part of the evaluation space. Changing all these parameters and including a wall resulted in 52% increase in error. Such increase in error when changing so many variables is considered as a success.

**Figure 16:**
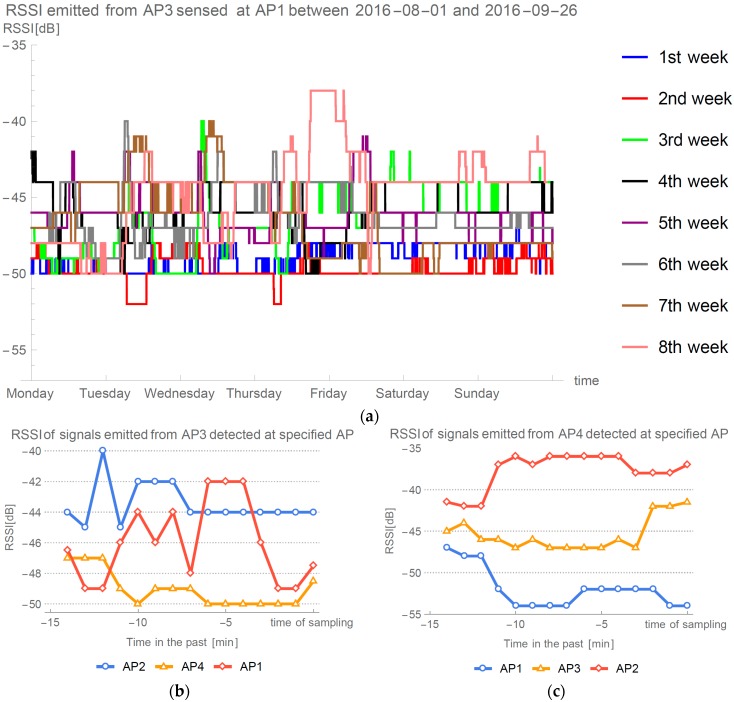
Analysis of unusual event on Thursday of the 8th week: (**a**) Analysis of RSSI from AP3 at AP1; (**b**) 15 min time frame of signals emitted from AP3 during unusual period; and (**c**) 15 min time frame of signals emitted from AP4 during unusual period.

Every Wi-Fi localization method is prone to errors when unexpected situations occur with the devices receiving or transmitting the signal. Researchers of modeled and fingerprint approaches try to avoid such situations during the evaluation. One such occurrence can be seen in Figure 5 on Thursday night of the 8th week. We can see a period of a few hours when the signals received by AP2 and emitted from AP4 have reached its maximum. Such events are usually handled by our method, but in this case similar thing occurred also on the connection between AP3 at AP1 as displayed in Figure 16a. Because we were present in the office at the time, we decided to investigate situation and preform sampling at each point of evaluation. 

**Figure 17:**
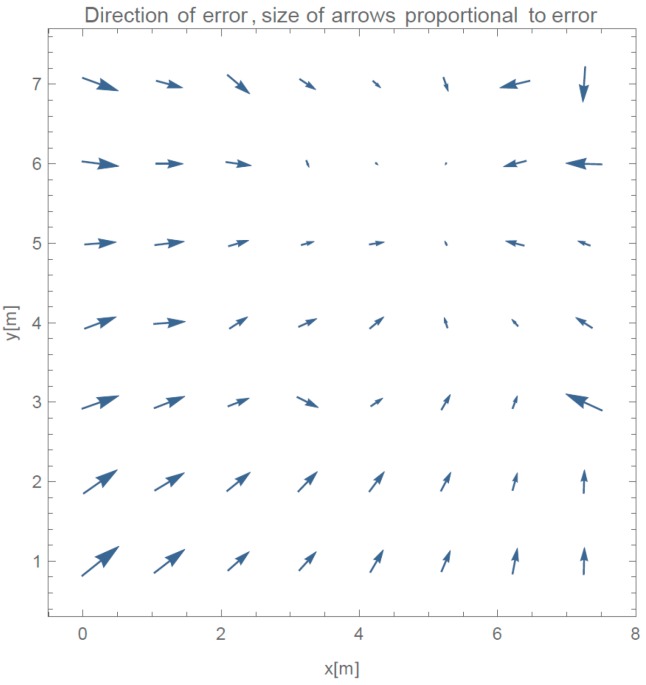
Error directions at the unusual event on Thursdays of the 8th week.

Figure 16b presents how signal emitted from the AP3 were detected at other three APs. Expected value for the AP1 is to be the least powerful, because it is positioned at the furthest position. Similarly, in Figure 16c, we would expect AP1 to have the highest RSSI, as it is the closest of the APs on the ideal place on the opposite wall. Such measurements of the signals resulted in negative values for exponent path loss parameter. Figure 17 presents the directions of the errors for the full evaluation, which we were able to perform during this event, without the implementation of the failback procedure discussed in Section 2.1.

Our method can detect similar events as it calculates unexpected (i.e., negative) values for γ. This is another advantage of our method in comparison to fingerprinting methods which cannot detect such situations. 

Implementing failback procedure on this dataset actually gave us great results. We obtained an average error of 2.43 m, and mean error of 2.00 m, which is number-wise better than our evaluation experiment. Because nearly 50% of γ values were replaced, no other significance should be given to these values other than our method gives usable results even in such circumstances. 

It is always difficult to compare the accuracy of the indoor localization methods. As methods are diverse, it is impossible to design a test set on which all the methods would be evaluated. Implementing multiple methods and evaluating them in common environment is the only possibility, but usually impossible to do due to hardware and software constrains. This usually originates from the authors of the papers, who often describe the evaluation environment vaguely. Data, other than the basic geometry, are often not written in the paper. We have tried to give as much detail as possible about the evaluation space to give the possibility of repeating the evaluation and create awareness that such information is necessary for even the simplest comparison. Another problem is that the accuracy of similar methods often depends greatly on the number of deployed APs, anchor points, beacons, etc. We tried to evaluate our method in suboptimal scenario and got average error between 2 and 3 m in static measuring conditions in single-room evaluation and 3 to 4 m in multi-room evaluation. Comparison of our results to the papers in Section 1 is heavily influenced by the information provided by the authors. Du et al. [31] report similar 2–3 m accuracy, but the results are difficult to compare, regarding different method requirements. Their main evaluation (and therefore majority of the evaluation points) is in a long narrow hallway, which is one of the places where path loss factor is lowest [34]. From their RSSI error it can be seen that RSSI error at points, which are not in the corridor (points numbered 15–25) have most variance and contain evaluation points with 10% of the biggest errors. Dumont et al. [30] report a mean error of 3–4 meters in an area approximately 4–5 times bigger than ours with 2.5 times more APs. Lim et al. [4] give great emphasis on number of APs involved into the localization procedure. From their final results it can be seen that when using 4 APs, median error of their approach is about 3.5 m in an experiment where mobile terminal was in AP mode and emitted signals towards APS for 2 min. Tarrío et al. [29] report similar errors in experiments with similar surface of interest. 

Our results confirm that our method achieves similar accuracy as other existing methods, therefore confirming that indoor Wi-Fi only localization method that does not require mobile terminal in access-point mode and does not require any additional hardware can be implemented. Our main scientific contribution is pure model-based Wi-Fi only approach that implements self-calibrating and self-adapting operability for real-world deployment. Our method considers the effects of architectural aspects (e.g., propagation loss through walls) and is applicable on widely available hardware. Our method requires multiple APs and a single localization server to run, thus making it ideal for applications in a variety of indoor situations. 

Our method has been developed with the main aim of providing localization for the IoT devices. Many of them will be deployed in our homes where there are no crowds of people, which would provide fingerprinting samples. Many devices are also stationary, with no usable data from IMU sensors. Although our method has been built with the localization of the IoT devices of the future in mind, it is applicable to different scenarios. Our method that does not saturate Wi-Fi channels by requiring terminals to be in AP mode, has great potential in heavily crowded places. Providing means of localization is in best interest of airports, commercial buildings, museums, etc. These buildings usually already have Wi-Fi infrastructure and offer mobile applications for visitors, which provide information, means of payments, ticketing, etc. Applications would benefit if indoor location could be determined and better information could be given to the users. Localization of tools and equipment in production halls is a common problem in the industry. Our method’s ability to adapt makes it a candidate for such deployments because of frequent movements of large metal objects with non-negligible impact on Wi-Fi propagation. When developing our method, we tried to keep hardware and software requirements simple in order for the method to be easily applicable and extendable. Although outside the scope of our research of pure Wi-Fi model-based approaches, simplicity of application of our proposed method gives researchers the opportunity to utilize our method and our propagation models in their own work. 

## 5. Conclusions

We have presented a novel localization method that was developed with the main aim of real-world applicability. Our proposed method is purely model-based and Wi-Fi only. It implements a continuous self-calibrating and self-adapting procedure insuring the adaption to the changes in Wi-Fi signal space. Our method is applicable on a variety of hardware and requires only the information regarding access points and walls. Parameters of modal propagation are inferred from the measurements by the method itself. Our method does not require mobile terminals to be in AP mode and thus not saturating Wi-Fi channels.

Our method utilizes well-known FSPL and ITU propagation models, which we have extended with additional parameters. First parameter describes the effect of direction of direct-signal-path in reference to the wall on which the emitting AP is mounted. Second parameter describes the effect of the walls on the propagation of the signal. 

To authors knowledge, this is the first Wi-Fi only indoor localization method that utilizes information about the architectural aspects, infers the parameters needed for the propagation simulation from measurements, continuously adapts to the indoor situation without human intervention, does not need any additional hardware beside the APs, and does not require mobile terminals to be in access-point mode. 

We have evaluated our method with great emphasis to real-world conditions and have chosen a realistic (suboptimal) environment for the evaluation. Evaluation has proven that our method achieves useful accuracy for indoor localization with average and median accuracy 2–3 and 3–4 m for single- and multi-room scenarios, respectively. This makes the results of our method comparable with the best other methods, of which the majority requires either more complex initial configuration or devices that are more sophisticated, or both. For justified interpretation of the evaluation results, it should also be noted that, in contrast to many other methods, we have chosen a realistic environment and not an optimized environment in which our method would perform even better.

In future research, we will extend the presented method by fusing the data gathered from IMU in order to develop a solution that will provide good results when tracking moving objects. Good underlying Wi-Fi only indoor localization method is a prerequisite for such approach. 

## Figures and Tables

**Table 1:** Comparison of the mean and median errors made by γ-only method.

Used RSSIs for γ Calculation	Median Error (m)	Average Error (m)	Standard Deviation (m)
γ-only—all APs	3.41	3.25	1.94
γ-only—omitted APs	2.51	2.64	1.43

**Table 2:** Comparison of the mean and median errors made by all three discussed methods.

Used RSSIs for γ Calculation	Median Error (m)	Average Error (m)	Standard Deviation (m)
γ-only—all APs	3.41	3.25	1.94
γ-only—omitted APs	2.51	2.64	1.43
Proposed method	2.29	2.63	1.45

**Table 3:** Comparison of the mean and median error made by all three discussed methods.

Evaluation of Proposed Method	Median Error (m)	Average Error (m)	Standard Deviation (m)
Single room	2.29	2.63	1.45
Multi room	3.48	3.22	1.58
